# The rehabilitation workforce in Brazil

**DOI:** 10.1186/s13690-024-01249-w

**Published:** 2024-02-26

**Authors:** Taciana Rocha dos Santos Sixel, Debora Bernardo, Arthur de Almeida Medeiros, Aylene Bousquat, Paulo Henrique dos Santos Mota, Ana Carolina Basso Schmitt

**Affiliations:** 1 Department of Physiotherapy, Speech Therapy and Occupational Therapy, Faculty of Medicine, University of São Paulo, São Paulo, SP Brazil; 2 Integrated Health Institute, Federal University of Mato Grosso do Sul, Campo Grande, MS Brazil; 3 Department of Politics, Management and Health, Faculty of Public Health, University of São Paulo, São Paulo, SP Brazil

**Keywords:** Workforce, Rehabilitation, Unified Health System, Health Professionals

## Abstract

**Background:**

The surge in individuals facing functional impairments has heightened the demand for rehabilitation services. Understanding the distribution of the rehabilitation workforce is pivotal for effective health system planning to address the population’s health needs.

**Objective:**

To investigate the spatial and temporal dispersion of physical therapists, speech therapists, psychologists and occupational therapists across various tiers of care within Brazil’s Unified Health System and its regions.

**Method:**

This is an ecological time series study on the supply of rehabilitation professionals. Data were obtained from the National Register of Health Establishments from 2007 to 2020. The density of professionals was calculated per 10,000 inhabitants annually for Brazil and its five regions. The Joinpoint regression model was used to analyze the temporal trends of the density of professionals, considering a 95% confidence interval.

**Results:**

In 2020, the most notable concentrations of psychologists, speech therapists, and occupational therapists in Brazil were observed in the domain of Specialized Health Care, with densities of 0.60, 0.20, and 0.16 professionals per 10,000 inhabitants, respectively. Conversely, the highest density of physical therapists was found within Hospital Health Care, with a density of 1.19 professionals per 10,000 inhabitants. Notably, variations in professional dispersion across different regions were apparent. Primary Health Care exhibited the highest density of professionals in the Northeast region, while the Southern region accounted for the highest densities in all professional categories within Specialized Health Care. The southeast region exhibited the largest workforce within Hospital Health Care. A marked upsurge in professional availability was noted across all categories, notably in the occupational therapy sector within hospital care (AAPC: 30.8), despite its initial low density.

**Conclusion:**

The implementation of public health policies played a significant role in the expansion of the rehabilitation workforce at all three levels of care in Brazil and its various regions from 2007 to 2020. Consequently, regional disparities and densities of professionals have emerged, mirroring patterns observed in low-income countries.

**Supplementary Information:**

The online version contains supplementary material available at 10.1186/s13690-024-01249-w.

**Table:** 

Text box 1. Contributions to the literature
• The analysis of the rehabilitation workforce using standardized working hours allows for a more accurate identification of the real scenario within this healthcare sector.
• Identifying the rehabilitation workforce across different levels of healthcare attention within the Unified Health System is a strong point of this article.
• The conclusions drawn in this article may be of interest to readers due to the emphasis on human resource management in the context of rehabilitation and public health.

## Introduction

The aging demographic, coupled with the surge in non-communicable chronic diseases, leads to an upsurge in the number of people with functional impairment, thus increasing the demand for rehabilitation [1]. In 2019, it was estimated that at least one in three people in the world (2.41 billion people) would require rehabilitation interventions at some point in their lives [2], and this scenario has certainly worsened with the Covid-19 pandemic and the emergence of long Covid. Rehabilitation comprises “a set of measures that help people with disabilities or about to acquire them to have and maintain optimal functioning in interaction with their environment [1]. It therefore reaches the entire population, including people who need temporary rehabilitation. It is essential for universal access to health care, permeating the different levels of care [1].

The literature indicates a limited capacity to meet the demand for rehabilitation, especially in low- and middle-income countries [3]. In Brazil, the Unified Health System also faces challenges to ensure access and provide the health care equipment and professionals needed for effective rehabilitation [4].

In response to the unmet rehabilitation needs worldwide, the World Health Organization (WHO) has promoted the Rehabilitation 2030 initiative: A Call for Action [5] in order to mobilize stakeholders to coordinated global action in priority areas, among which are: building comprehensive service delivery models in rehabilitation, expanding the rehabilitation workforce, and improving data collection.

The workforce is one of the issues that needs to be addressed in meeting rehabilitation needs. It is worth noting that the list of professions that make up the rehabilitation workforce varies between countries, and even within the same country [6]. Among the most commonly included professional categories are: physical therapists, speech therapists, psychologists, and occupational therapists [1]. Assessing the workforce capacity and dispersion of these professionals across different levels of care facilitates the development of public health policies aimed at promoting health and preventing diseases, thereby enhancing the provision of comprehensive care [1, 3].

Existing research on Brazil’s healthcare workforce predominantly focuses on physicians and nurses, offering limited insights into the rehabilitation workforce and its distribution within the healthcare network. Based on the principle that “there is no health without workforce” [7] and that rehabilitation has been considered an important health demand of the 21st century, it is essential to know the rehabilitation workforce to better plan the health system. Consequently, the present study aims to identify the spatial-temporal distribution of physical therapists, speech therapists, psychologists, and occupational therapists across the levels of health care of the Brazilian Unified Health System from 2007 to 2020.

## Methods

This study constitutes an ecological analysis of longitudinal data, focusing on the availability of rehabilitation professionals within Brazil’s Unified Health System. Specifically, the investigation encompasses the following professional categories: physical therapists, speech therapists, psychologists, and occupational therapists.

The dataset was obtained from the *Cadastro Nacional de Estabelecimentos de Saúde* (CNES), available on the website of the *Departamento de Informática do Sistema Único de Saúde do Brasil* (DATASUS) (http://cnes.datasus.gov.br). Data extraction and pre-processing were performed in the RStudio 1.2 software and the microdatasus [8] package, from the CNES-PF database.

The dataset was collected from 2007 (when the occupations were updated by the Brazilian Classification of Occupations at DATASUS) to 2020, considering August as the reference point for each year analyzed. It is recommended to analyze the workforce for a minimum period of 10 years to observe long-term actions with sustainable results in its expansion [7]. The analyses about the supply of occupational therapists in the country were carried out starting in 2009, when the records of this occupation began in the information systems.

For this study, the levels of care considered were: Primary Health Care (PHC), Specialized Health Care (SHC) and Hospital Health Care (HHC). Each level of care encompassed distinct services categorized in the National Registry of Health Establishments as follows:

- 1) PHC: health post, health center/basic unit, mixed unit, land mobile unit, river mobile unit, family health support center, health academy pole, isolated home care service (Home Care) residential care unit.
- 2) SHC: polyclinic, isolated office, clinic/specialty center, psychosocial care center, orthopedic workshop.
- 3) HHC: general hospital, specialized hospital, isolated day hospital [9].

In Brazil, there is specific legislation for some professional categories regarding work hours, such as physiotherapy and occupational therapy [10], in addition to the possibility of several links and different workloads, which directly impacts the number of professionals working in health services. Therefore, to correct this bias, the workload was standardized to 40 h a week [11].

Next, the density of professionals per 10,000 inhabitants was calculated, considering the workforce in rehabilitation, by occupation and by year, divided by the total population of Brazil and geographic region, multiplying by 10,000 inhabitants. The estimate of the Brazilian population from 2007 to 2020 was obtained from the Brazilian Institute of Geography and Statistics. For this analysis, the indicator “Density of active health workers per 10,000 population at subnational level” [12] was taken as the basis.

The temporal trend analysis of the density of rehabilitation professionals per year in Brazil and geographic regions was performed. Regression analysis was carried out in the software Joipoint Regression Program version 4.7.0, in which the average annual percentage variation was estimated, considering a confidence interval of 95%. The final model chosen was the best fit, with the Annual Percentage Change (APC) based on the trend of each segment, identifying whether these values were statistically significant (*p* < 0.05). In order to quantify the trend of the years studied, the Average Annual Pencentage Change (AAPC) was calculated, based on the cumulative geometric mean of the APC trends, with equal weights for the lengths of each segment during the fixed interval. Significance testing was based on the Monte Carlo permutation method and the calculation of the annual percentage change in the ratio using the logarithm of the ratio [13, 14].

The research was approved by the Research Ethics Committee of the University of São Paulo Medical School under opinion no. 283/18.

## Results

In Brazil, the spatial and temporal distribution of health professionals showed variation among the categories in the levels of health care. A significant increase in the work force of physical therapists, speech therapists, psychologists and occupational therapists was observed at all levels of care in the period analyzed. Occupational therapists represent the professional category that showed the greatest growth (PHC: 30.8), despite an initially low density of professionals (Table 1).

**Table 1:** Time trend of the workforce of rehabilitation professionals per 10,000 inhabitants in Primary Health Care, Specialized Health Care and Hospital Health Care. Brazil, 2007 to 2020

	Seg.	**Primary Health Care**				**Specialized Health Care**				**Hospital Health Care**			
		Start Year (dens.)	End Year (dens.)	APC	AAPC	Start Year (dens.)	End Year (dens.)	APC	AAPC	Start Year (dens.)	End Year (dens.)	APC	AAPC
**Physiotherapy**	1	2007 (0,12)	2009 (0,20)	26,3^a^	10,8^a^	2007 (0,25)	2010 (0,36)	12,9^a^	5,4^a^	2007 (0,26)	2009 (0,39)	25,2^a^	11,3^a^
	2	2009 (0,20)	2014 (0,36)	12,4^a^		2010 (0,36)	2020 (0,52)	3,3^a^		2009 (0,39)	2020 (1,19)	8,9^a^	
	3	2014 (0,36)	2020 (0,46)	4,7^a^		-	-	-	-	-	-	-	-
**Speech Therapy**	1	2007 (0,05)	2010 (0,09)	21,5^a^	7,6^a^	2007 (0,12)	2010 (0,16)	9,6^a^	3,5^a^	2007 (0,05)	2009 (0,07)	20,3^a^	9,4
	2	2010 (0,09)	2018 (0,15)	5,4^a^		2010 (0,16)	2020 (0,20)	1,8^a^		2009 (0,07)	2013 (0,10)	9,9^a^	
	3	2018 (0,15)	2020 (0,14)	-2,4		-	-	-	-	2013 (0,10)	2020 (0,16)	6,1^a^	
**Psycology**	1	2007 (0,20)	2014 (0,37)	8,7^a^	6,8^a^	2007 (0,29)	2010 (0,41)	11,7^a^	5,3^a^	2007 (0,16)	2010 (0,21)	9,6^a^	5,8^a^
	2	2014 (0,37)	2020 (0,47)	4,6^a^		2010 (0,41)	2020 (0,60)	3,4^a^		2010 (0,21)	2020 (0,35)	4,7^a^	
**Occupational Therapy**	1	2009 (< 0,01)	2011 (0,03)	286,1^a^	28,3^a^	2009 (< 0,01)	2011 (0,13)	310,8^a^	27,4^a^	2009 (< 0,01)	2011 (0,06)	357,5^a^	30,8^a^
	2	2011 (0,03)	2020 (0,04)	0,4		2011 (0,13)	2020 (0,16)	-1,8^a^		2011 (0,06)	2020 (0,07)	-1,0^a^	

*Seg* Segments with the best statistical adjustments, *Initial Year* Initial year of the segment, *Final Year* Final year of the segment, *Dens* Professional density per 10,000 inhabitants, *APC* Annual Percent Change, *AAPC* Average Annual Percent Change, *95%* 95% confidence interval

^a^Statistically significant at the 5% level

In PHC, the density of psychologists rose from 0.20 per 10,000 inhabitants in 2007 to 0.47 in 2020, and of physical therapists from 0.12 per 10,000 inhabitants to 0.46, an increase of 135% and 283.3%, respectively Analyzing the Brazilian regions in 2020, the Northeast region emerged with the highest density figures for physical therapists (0.63/10,000 inhabitants), speech therapists (0.16/10,000 inhabitants), and occupational therapists (0.05/10,000 inhabitants). Conversely, the Southern region displayed the highest density of psychologists (0.59/10,000 inhabitants) (Table 2). In SHC, there was an increase in the density of all professional categories, with more expressive results for psychologists, whose density went from 0.29 per 10,000 inhabitants in 2007, to 0.60 in 2020, representing an increase of 106.9%. The Southern region presented the highest density of professionals during the entire period analyzed (Table 2). In HHC, the highest density observed in Brazil was of physical therapists from 0.26 per 10,000 inhabitants in 2007 to 1.19 in 2020, an increase of 357.7%. When observing the Brazilian regions, throughout the period examined, the Southeast had the highest density in all professional categories, but in 2020, the Midwest region had the highest growth (1.31 physical therapists, 0.41 psychologists, 0.19 speech therapists and 0.09 occupational therapists) (Table 2). An additional table show this in more detail (see Additional file 1).

**Table 2:**
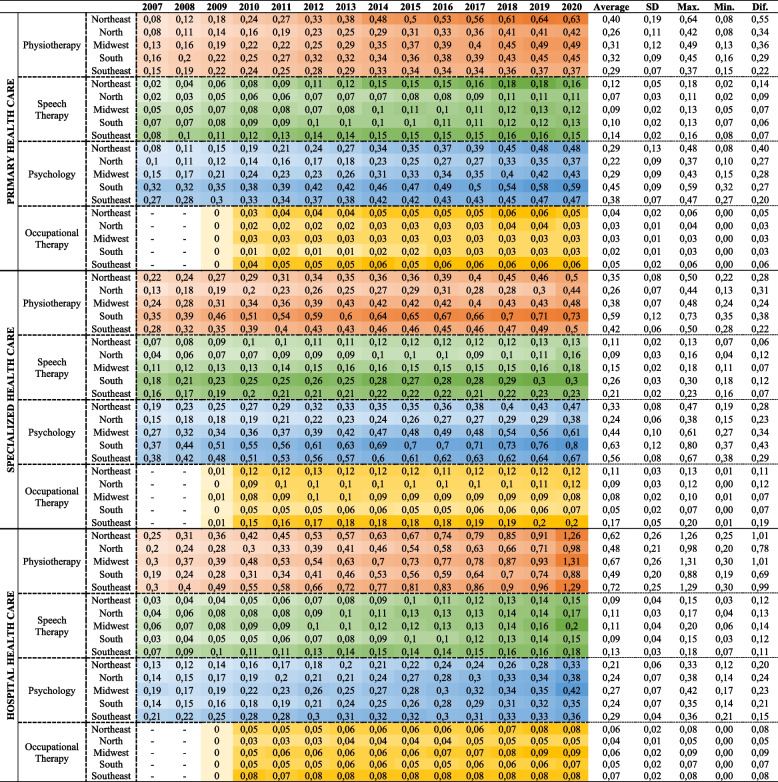
Density of rehabilitation professionals per 10,000 inhabitants in Primary Health Care, Specialized Health Care, and Hospital Health Care in Brazilian regions. Brazil, 2007 to 2020

*SD* Standard Deviation, *Max* Maximum, *Min* Minimum, *Dif* Diferrence

In PHC, the highest growth results were in the Northeast: occupational therapists (AAPC: 29.9), speech therapists (AAPC: 18.2), physical therapists (AAPC: 17.7), and psychologists (AAPC: 14.9). Regarding the SHC and HHC, the biggest growth varied between two regions. In SHC, the most expressive results for occupational therapists (AAPC: 34.1) and speech therapists (AAPC: 9.4) were in the North region, psychologists (AAPC: 6.5) in the Northeast region, and physical therapists (AAPC: 6.4) in the North and Northeast regions. In HHC, greater growth was observed for occupational therapists (AAPC: 34.9), speech therapists (AAPC: 13.1) and physical therapists (AAPC: 11.9) in the Northeast region, while for psychologists (AAPC: 7.8) in the North region (Table 3).

**Table 3:** Time trend of the workforce of rehabilitation professionals per 10,000 inhabitants in Primary Health Care, Specialized Health Care, and Hospital Health Care in Brazilian regions. Brazil, 2007 to 2020

	Seg.	**Midwest**				**Northeast**				**North**				**Southeast**				**South**			
		Start Year	End Year	APC	AAPC	Start Year	End Year	APC	AAPC	Start Year	End Year	APC	AAPC	Start Year	End Year	APC	AAPC	Start Year	End Year	APC	AAPC
**Primary Health Care**																					
Physiotherapy	1	2007	2014	13,7^a^	10,5^a^	2007	2009	54,3^a^	17,7^a^	2007	2009	30,3^a^	13,6^a^	2007	2009	19,6^a^	7,1^a^	2007	2012	12,9^a^	7,9^a^
	2	2014	2020	6,8^a^		2009	2014	20,1^a^		2009	2014	15,5^a^		2009	2014	7,9^a^		2012	2020	4,9^a^	
	3	-	-	-	-	2014	2020	5,8^a^		2014	2020	7,0^a^		2014	2020	2,6^a^		-	-	-	-
Speech Therapy	1	2007	2010	18,1^a^	8,0^a^	2007	2009	76,1^a^	18,2^a^	2007	2010	36,5^a^	12,9^a^	2007	2010	15,1^a^	4,7^a^	2007	2010	9,8^a^	4,9^a^
	2	2010	2020	5,1^a^		2009	2014	19,1^a^		2010	2020	6,7^a^		2010	2018	3,0^a^		2010	2020	3,5^a^	
	3	-	-	-	-	2014	2020	2,9		-	-	-	-	2018	2020	-3,0		-	-	-	-
Psychologi	1	2007	2020	8,0^a^	8,0^a^	2007	2009	38,4^a^	14,9^a^	2007	2020	11,0^a^	11,0^a^	2007	2014	6,1^a^	4,3^a^	2007	2020	5,0^a^	5,0^a^
	2	-	-	-	-	2009	2014	16,1^a^		-	-	-	-	2014	2020	2,3^a^		-	-	-	-
	3	-	-	-	-	2014	2020	7,0^a^		-	-	-	-	-	-	-	-	-	-	-	-
Occupational Therapy	1	2009	2011	218,5^a^	21,8^a^	2009	2011	309,2^a^	29,9^a^	2009	2020	19,2^a^	19,2^a^	2009	2011	295,4^a^	27,1^a^	2009	2020	26,7^a^	26,7^a^
	2	2011	2020	-1,6		2011	2020	0,7		-	-	-	-	2011	2020	-1,2^a^		-	-	-	-
**Specialized Health Care**																					
Physiotherapy	1	2007	2013	9,6^a^	4,9^a^	2007	2012	8,9^a^	6,4^a^	2007	2020	6,4^a^	6,4^a^	2007	2010	12,9^a^	4,6^a^	2007	2010	13,8^a^	5,8^a^
	2	2013	2017	-1,6		2012	2015	2,3		-	-	-	-	2010	2020	2,2^a^		2010	2014	5,4^a^	
	3	2017	2020	4,8		2015	2020	6,3^a^		-	-	-	-	-	-	-	-	2014	2020	2,2^a^	
Speech Therapy	1	2007	2013	6,0^a^	3,6^a^	2007	2010	12,8^a^	5,0^a^	2007	2011	16,9^a^	9,4^a^	2007	2010	8,4^a^	2,7^a^	2007	2009	14,9^a^	4,1^a^
	2	2013	2018	-1,7^a^		2010	2020	2,7^a^		2011	2018	1,8		2010	2020	1,1^a^		2009	2020	2,3^a^	
	3	2018	2020	10,4^a^		-	-	-	-	2018	2020	23,5		-	-	-	-	-	-	-	-
Psychologi	1	2007	2020	5,7^a^	5,7^a^	2007	2010	12,0^a^	6,5^a^	2007	2020	6,1^a^	6,1^a^	2007	2009	12,5^a^	4,2^a^	2007	2009	17,6^a^	5,9^a^
	2	-	-	-	-	2010	2020	4,9^a^		-	-	-	-	2009	2014	4,7^a^		2009	2014	5,7^a^	
	3	-	-	-	-	-	-	-	-	-	-	-	-	2014	2020	1,2^a^		2014	2020	2,5^a^	
Occupational Therapy	1	2009	2011	233,8^a^	20,8^a^	2009	2011	253,3^a^	22,6^a^	2009	2011	456,0^a^	34,1^a^	2009	2011	366,0^a^	30,9^a^	2009	2011	290,9^a^	26,7^a^
	2	2011	2020	-3,6^a^		2011	2020	-3,1^a^		2011	2020	-2,3^a^		2011	2020	-1,3^a^		2011	2020	-1,4^a^	
**Hospital Health Care**																					
Physiotherapy	1	2007	2014	12,1^a^	11,1^a^	2007	2010	18,1^a^	11,9^a^	2007	2020	11,4^a^	11,4^a^	2007	2009	30,0^a^	10,6^a^	2007	2013	15,3^a^	11,6^a^
	2	2014	2018	3,8		2010	2020	10,1^a^		-	-	-	-	2009	2020	7,4^a^		2013	2020	8,5^a^	
	3	2018	2020	23,5^a^		-	-	-	-	-	-	-	-	-	-	-	-	-	-	-	-
Speech Therapy	1	2007	2009	18,3^a^	9,8^a^	2007	2013	18,4^a^	13,1^a^	2007	2009	28,8^a^	10,4^a^	2007	2009	23,2^a^	7,4^a^	2007	2012	17,5^a^	12,5^a^
	2	2009	2018	6,3^a^		2013	2020	8,7^a^		2009	2020	7,3^a^		2009	2020	4,8^a^		2012	2020	9,4^a^	
	3	2018	2020	17,6^a^		-	-	-	-	-	-	-	-	-	-	-	-	-	-	-	-
Psychologi	1	2007	2020	6,3^a^	6,3^a^	2007	2020	7,5^a^	7,5^a^	2007	2020	7,8^a^	7,8^a^	2007	2010	10,5^a^	4,1^a^	2007	2014	8,5^a^	7,1^a^
	2	-	-	-	-	-	-	-	-	-	-	-	-	2010	2020	2,3^a^		2014	2020	5,5^a^	
Terapia Ocupacional	1	2009	2011	249,8^a^	28,7^a^	2009	2011	393,2^a^	34,9^a^	2009	2011	370,6^a^	35,2^a^	2009	2011	438,1^a^	32,0^a^	2009	2011	224,9^a^	23,8^a^
	2	2011	2020	3,1^a^		2011	2020	1,1		2011	2020	2,5^a^		2011	2020	-3,4^a^		2011	2020	-0,1	

*Segment* Segments according to the best statistical fit, *Initial Year* Initial year of the segment, *Final Year* Final year of the segment, *Dens* Professional density per 10,000 inhabitants, *APC* Annual Percent Change, *AAPC* Average Annual Percent Change, *95% CI* 95% confidence interval

^a^Statistically significant at the 5% level

## Discussion

Understanding the dynamics behind the growth of the rehabilitation workforce across Brazil’s three levels of care becomes pivotal as the demand for rehabilitation escalates. This comprehension encompasses insights into professionals’ availability, skill sets, motivations, performance, and geographical distribution [15].

There is no official recommendation on the ideal number of professionals to provide comprehensive care in rehabilitation6. However, Rehabilitation 2030: A Call for Action [5] pointed out that there is a shortage of qualified professionals, and thus rehabilitation care remains underserved.

Workforce needs and demands for rehabilitation are different in each local context. The capacity of the rehabilitation workforce in high-income countries is greater than in low- and middle-income countries. And even among high-income countries, the supply of professionals is highly variable, differing up to 40 times [16]. Jesus et al. reported densities of rehabilitation professionals ranging from < 0.01 per 10,000 population in low-income countries to up to 25 per 10,000 population in high-income countries [3]. Our results indicate densities of professionals in Brazil in 2020 closer to the rates of low-income countries.

In this study, we observed that the highest density of physical therapists in 2020 in Brazil was 2.17 professionals per 10,000 inhabitants. Different from the density in Ireland, which was 6.8 [17], but close to Canada, of 2.32 [18], and higher than Singapore, with 1.8 physiotherapists per 10,000 inhabitants [6]. As for psychologists, the density observed for Brazil was 1.42 professionals per 10,000 inhabitants, while the United States had 3.0 per 10,000 inhabitants [19]. The supply of speech therapists was 0.50 speech therapists per 10,000 inhabitants, values adjacent to South Africa [20] with a density of 0.57. As for occupational therapists, the density was 0.27 per 10,000 inhabitants, lower than the average of 0.9 in South Africa [21], 1.9 in Portugal [6] and 3.6 in the United States [6]. It is worth noting that this study considered only the professionals who provide services in health facilities of the Unified Health System.

The diversity within the rehabilitation workforce is accompanied by variability in practice scope and non-uniformity in data collection across studies, with some instances lacking available data [6, 16], Notably, most studies conducted their analyses based on absolute professional numbers without organization by healthcare levels, underscoring the importance of studies that delineate the specific placements of these professionals within the healthcare system. Such studies should standardize by workload to facilitate user access.

Within PHC, in the period studied, there was a significant upswing in rehabilitation professionals. A pivotal policy, likely contributing to this surge, was the creation of the Extended Family Health Center [22] that expanded the professional actions of primary care, including rehabilitation. The federal financial incentive to implement these teams - and the consequent expansion of PHC jobs - may have directly impacted the growth and supply of different professions that can compose the PHC teams, such as physical therapists, speech therapists, psychologists, and occupational therapists. However, the reorientation of the work process of the teams, introduced by the National Primary Care Policy [23] of 2017, as well as the change in funding in PHC, by Previne Brazil [24] in 2019, went in the opposite direction, and may compromise the expansion in the rehabilitation workforce. In this aspect, the findings of the study already indicate a reduction in the density of speech therapists as of 2018, as well as stabilization in the density of occupational therapists and deceleration in the growth of the other categories. These shifts in professional density might already reflect the impacts of these policies.

Between 2007 and 2020, the notable rise in the numbers of physical therapists, speech therapists, psychologists, and occupational therapists across the three levels of care reflects not just network expansion but also its increasing complexity. It underscores the establishment of policies specifically targeting care enhancement, such as the Care Network for People with Disabilities [25]. This network aimed to broaden access and improve the quality of care for individuals with diverse types of disabilities, whether temporary or permanent, progressive, regressive, or stable, addressing various disabilities, including those related to hearing, physical, intellectual, visual impairments, ostomy, and multiple disabilities.

The workforce growth, especially in SHC, peaked around 2012, the year of the important launch of two public policies that induce the creation and strengthening of specialized strategic services: the Specialized Rehabilitation Center for specialized outpatient care in rehabilitation that performs diagnosis, treatment, granting, adaptation and maintenance of assistive technology; and the Psychosocial Care Center, catering to people with psychic suffering or mental disorders, including those with needs arising from the use of alcohol, crack and other substances, who are in crisis situations or in psychosocial rehabilitation processes, as essential components of the Care Network for People with Disabilities [25] and Psychosocial Care Network [26].

Historically, rehabilitation actions have been executed within SHC [1]. This study underscores that, during the period analyzed, there was a greater concentration of professionals at this level of care, except for the physical therapists, who showed greater concentration in HHC. Rodes et al [4] also observed this situation in the period 2007–2015 and pointed out as a possible influence the Resolution nº 7, of February 24, 2010 [27], which establishes minimum standards for the functioning of Intensive Care Units, imposing the performance of at least one physiotherapist for every ten beds, 18 h a day. Among the benefits of the work of the physiotherapist in this area, are the reduction of mechanical ventilation time and the length of patient stay, reducing hospital costs [28]. The increase of physiotherapists in 2020 in HHC coincides with the first wave of Covid-19 deaths in Brazil. In that environment, the physiotherapist was fundamental to guarantee the monitoring of the patient’s respiratory mechanics.

The regional distribution of the rehabilitation workforce in Brazil presents disparities across different care levels. In Primary Health Care (PHC), the Northeast region displayed the highest densities of physical therapists, speech therapists, and occupational therapists. This can be attributed to its status as the region with the most comprehensive and well-distributed Family Health, Oral Health, and Expanded Family Health teams [29]. The expansion of rehabilitation actions in PHC is essential for long-term care, contributing to improve the quality of life of the population [2].

Regarding SHC, the Southern region concentrated the highest densities in all professional categories, while in PHC, the Southeast region had the largest workforce. The higher concentration of professionals in the South and Southeast is a historical issue in the country, justified by several factors, notably because they are areas with greater concentration of resources, technology, and educational institutions [30].

It is worth noting that inequalities in the geographic distribution of services and of the workforce is a worldwide problem, which demands strategies for the recruitment and retention of professionals to guarantee universal access to the population [31].

Public policies can direct more resources for the development of the health workforce. However, as pointed out by Silva et al. [32], it can be detrimental to witness an expansion in care provision followed by measures that undermine these efforts. For instance, the MS/GM Ordinance No. 3992/2017 [33] altered the transfer of resources for public health services, reducing funding blocks within the Unified Health System from six to two. Such shifts pose risks to both service and human resources expansion within the healthcare sector.

As a limitation of this study, we point out the use of secondary data with the possibility of incomplete information in the CNES [34], but this is the official database of the Brazilian Ministry of Health. The analyses were relativized for a weekly workload of 40 h, known as Full Time Equivalent, since professionals can have different workloads. Its use is beneficial in planning, monitoring, and comparing the health workforce, including between countries [35]. We specifically considered four professions that contribute to rehabilitation in the public health sector, but the insertion of other professional categories could broaden the discussion of health care in this area. Due to the absence of a current census, the estimated population was used based on data from the 2010 census.

Regional differences in the workforce compromise access to rehabilitation care for the most vulnerable populations. Although there is not a preconized number for the size of the workforce, studies point to the need to expand the supply of skilled labor in rehabilitation care [5]. In this sense, the identification of the rehabilitation workforce in Brazil is important to understand the distribution of professionals and what has occurred over the years. Moreover, the results may contribute to the planning of care management in rehabilitation, in order to provide better care and access to health services for the population.

## Final considerations

Public health policies contributed to the growth of the rehabilitation workforce across three levels of care throughout Brazil from 2007 to 2020. The increase in population needs for rehabilitation services and the increase in the supply of professionals in the job market may also have contributed to this growth. In Brazil, the density of rehabilitation professionals in the Unified Health System is similar to low-income countries. Significantly, regional disparities were evident, with greater concentration of professionals in PHC in the Northeast region, in SHC in the Southern region, and in HHC in the Southeast region.

### Supplementary Information

**Additional file 1: Supplementary table 1.** Time trend density of rehabilitation professionals per 10,000 inhabitants in Primary Health Care, Specialized Health Care, and Hospital Health Care in Brazilian regions. Brazil, 2007 to 2020.

## Data Availability

The datasets used and/or analyzed during the current study are available from the corresponding author on reasonable request. However, the original data was obtained from the CNES, available on the DATASUS website. http://www2.datasus.gov.br/DATASUS/index.php?area=0204&id=6906 Accessed 10 March 2020. Data was updated in 2021 to include 2020 values.

## References

[1] World Health Organization (2011). World report on disability.

[2] Cieza A, Causey K, Kamenov K, Hanson SW, Chatterji S, Vos T (2021). Global estimates of the need for rehabilitation based on the Global Burden of Disease study 2019: a systematic analysis for the Global Burden of Disease Study 2019. Lancet.

[3] Jesus TS, Landry MD, Dussault G, Fronteira I (2017). Human resources for health (and rehabilitation): Six Rehab-Workforce Challenges for the century. Hum Resour Health.

[4] Rodes CH, Kurebayashi R, Kondo VE, Luft VD, de GóesÂngela B, Schmitt ACB (2017). O acesso e o fazer da reabilitação na Atenção Primária à Saúde. Fisioter Pesqui.

[5] World Health Organization. Rehabilitation 2030: a call for action: 6–7 February 2017, Executive Boardroom, WHO Headquarters, meeting report. WHO; 2020. Available from: https://apps.who.int/iris/handle/10665/339910.

[6] Jesus TS, Koh G, Landry M, Ong P-H, Lopes AMF, Green PL (2016). Finding the “right-size” physical therapy workforce: international perspective across 4 countries. Phys Ther.

[7] World Health Organization (2014). A universal truth: no health without a workforce.

[8] Saldanha RDF, Bastos RR, Barcellos C. Microdatasus: pacote para download e pré-processamento de microdados do Departamento de Informática do SUS (DATASUS). Cad. de Saúde Pública. 2019;35(9). 10.1590/0102-311X00032419. 10.1590/0102-311X00032419 31531513

[9] Brasil. Ministério da Saúde. Tipo de estabelecimento. DATASUS; 2020. Available from: http://tabnet.datasus.gov.br/cgi/cnes/tipo_estabelecimento.htm.

[10] Brasil. Lei nº. 8.856, 1º de março de 1994. Fixa a jornada de trabalho dos profissionais fisioterapeuta e terapeuta ocupacional. Available from http://www.planalto.gov.br/ccivil_03/leis/l8856.htm. Accessed 14 Oct 2022.

[11] Universidade Federal de Minas Gerais. Construção do índice de escassez de profissionais de saúde para apoio à Política Nacional de Promoção da Segurança Assistencial em Saúde. 2010. Available from http://epsm.nescon.medicina.ufmg.br/epsm/Relate_Pesquisa/Index_relatorio.pdf. Citado 18 de agosto de 2022.

[12] World Health Organization (2017). National health workforce accounts: a handbook.

[13] Kim HJ, Fay MP, Feuer EJ, Midthune DN (2000). Permutation tests for joinpoint regression with applications to cancer rates. Stat Med.

[14] Kim HJ, Fay MP, Yu B, Barrett MJ, Feuer EJ (2004). Comparability of segmented line regression models. Biometrics.

[15] World Health Organization (2021). Health labour market analysis guidebook.

[16] Conradie T, Berner K, Louw Q. Rehabilitation workforce descriptors: a scoping review. BMC Health Serv Res. 2022;22(1). 10.1186/s12913-022-08531-z. 10.1186/s12913-022-08531-z PMC9482289 36115976

[17] Eighan J, Walsh B, Smith S, Wren MA, Barron S, Morgenroth E (2018). A profile of physiotherapy supply in Ireland. Ir J Med Sci.

[18] Shah TI, Milosavljevic S, Trask C, Bath B (2019). Mapping physiotherapy use in Canada in relation to physiotherapist distribution. Physiother Can.

[19] Andrilla CHA, Patterson DG, Garberson LA, Coulthard C, Larson EH (2018). Geographic variation in the supply of selected behavioral health providers. Am J Prev Med.

[20] Pillay M, Tiwari R, Kathard H, Chikte U. Sustainable workforce: South African audiologists and speech therapists. Hum Resour Health. 2020;18(1). 10.1186/s12960-020-00488-6. 10.1186/s12960-020-00488-6 PMC7329495 32611357

[21] Ned L, Tiwari R, Buchanan H, Van Niekerk L, Sherry K, Chikte U. Changing demographic trends among South African occupational therapists: 2002 to 2018. Hum Resour Health. 2020;18(1). 10.1186/s12960-020-0464-3. 10.1186/s12960-020-0464-3 PMC7083000 32192502

[22] Ministério da Saúde (BR), Gabinete do Ministro. Portaria Nº. 154, de 24 de janeiro de 2008. Cria os Núcleos de Apoio à Saúde da Família - NASF. Available from https://bvsms.saude.gov.br/bvs/saudelegis/gm/2008/prt0154_24_01_2008.html. Accessed 18 Oct 2022.

[23] Ministério da Saúde (BR), Gabinete do Ministro. Portaria Nº 2.436, de 21 de setembro de 2017. Aprova a Política Nacional de Atenção Básica, estabelecendo a revisão de diretrizes para a organização da Atenção Básica, no âmbito do Sistema Único de Saúde (SUS). Available from https://bvsms.saude.gov.br/bvs/saudelegis/gm/2017/prt2436_22_09_2017.html. Accessed 18 Oct 2022.

[24] Ministério da Saúde (BR), Gabinete do Ministro. Portaria Nº 2.979, de 12 de novembro de 2019. Institui o Programa Previne Brasil, que estabelece novo modelo de financiamento de custeio da Atenção Primária à Saúde no âmbito Sistema Único de Saúde, por meio da alteração da Portaria de Consolidação no 6/GM/MS, de 28 de setembro de 2017. Diário Oficial da União 13 nov 2019;Seção:1. Available from https://bvsms.saude.gov.br/bvs/saudelegis/gm/2019/prt2979_13_11_2019.html. Accessed 23 Oct 2022.

[25] Ministério da Saúde (BR), Gabinete do Ministro. Portaria Nº 793, de 24 de abril de 2012. Institui a Rede de Cuidados à Pessoa com Deficiência no âmbito do Sistema Único de Saúde. Available from https://bvsms.saude.gov.br/bvs/saudelegis/gm/2012/prt0793_24_04_2012.html. Accessed 14 Oct 2022.

[26] Ministério da Saúde (BR), Gabinete do Ministro. Portaria nº 3.088, de 23 de dezembro de 2011. Institui a Rede de Atenção Psicossocial para pessoas com sofrimento ou transtorno mental e com necessidades decorrentes do uso de crack, álcool e outras drogas, no âmbito do Sistema Único de Saúde (SUS). Diário Oficial da União 23 dez 2011. Available from https://bvsms.saude.gov.br/bvs/saudelegis/gm/2011/prt3088_23_12_2011_rep.html. Accessed 24 Oct 2022.

[27] Ministério da Saúde (BR), Agência Nacional de Vigilância Sanitária. Resolução nº 7, de 24 de fevereiro de 2010. Dispõe sobre os requisitos mínimos para funcionamento de Unidades de Terapia Intensiva e dá outras providências. Diário Oficial da União. 25 fev 2010; Seção:1. Available from https://bvsms.saude.gov.br/bvs/saudelegis/anvisa/2010/res0007_24_02_2010.html.

[28] Rotta BP, da Silva JM, Fu C, Goulardins JB, Pires-Neto RDC, Tanaka C (2018). Relationship between availability of physiotherapy services and ICU costs. J Bras Pneumol.

[29] SoaresFilho AM, Vasconcelos CH, Dias AC, de Souza ACC, Merchan-Hamann E, da Silva MRF (2022). Atenção Primária à Saúde no Norte e Nordeste do Brasil: mapeando disparidades na distribuição de equipes. Ciênc Saúde Coletiva.

[30] de Albuquerque MV, Viana AL d’Ávila, de Lima LD, Ferreira MP, Fusaro ER, Iozzi FL. Desigualdades regionais na saúde: mudanças observadas no Brasil de 2000 a 2016. Ciênc Saúde Coletiva. 2017;22(4):1055–64. 10.1590/1413-81232017224.26862016. Citado 24 de outubro de 2022. 10.1590/1413-81232017224.26862016 28444033

[31] World Health Organization (2016). Global strategy on human resources for health: workforce 2030.

[32] da Silva DB, Dos Santos Sixel TR, de Almeida MA, Dos Santos Mota PH, Bousquat A, Schmitt ACB (2021). The workforce for rehabilitation in primary health care in Brazil. Hum Resour Health.

[33] Ministério da Saúde (BR), Gabinete do Ministro. Portaria nº 3992, de 28 de dezembro de 2017. Altera a Portaria de Consolidação nº 6/ GM/MS, de 28 de setembro de 2017, para dispor sobre o financiamento e a transferência dos recursos federais para as ações e os serviços públicos de saúde do Sistema Único de Saúde. Available from https://bvsms.saude.gov.br/bvs/saudelegis/gm/2017/prt3992_28_12_2017.html. Accessed 14 Oct 2022.

[34] Rocha TAH, da Silva NC, Barbosa ACQ, Amaral PV, Thumé E, Rocha JV (2018). Cadastro Nacional de Estabelecimentos de Saúde: evidências sobre a confiabilidade dos dados. Ciênc Saúde Coletiva.

[35] Girasek E, Kovács E, Aszalós Z, Eke E, Ragány K, Kovács R, et al. Headcount and FTE data in the European health workforce monitoring and planning process. Hum Resour Health. 2016;14(1). 10.1186/s12960-016-0139-2. 10.1186/s12960-016-0139-2 PMC4947240 27423330

