# Supplementary material for: The rehabilitation workforce in Brazil
Source: Arch Public Health. 2024 Feb 26;82:25. doi: 10.1186/s13690-024-01249-w (PMC10895827; doi:10.1186/s13690-024-01249-w)
Supplement: Supplementary file 1 — Additional file 1: Supplementary table 1. Time trend density of rehabilitation professionals per 10,000 inhabitants in Primary Health Care, Specialized Health Care, and Hospital Health Care in Brazilian regions. Brazil, 2007 to 2020. [file 13690_2024_1249_MOESM1_ESM.pdf]

Supplementary table 1. Time trend density of rehabilitation professionals per 10,000 inhabitants in Primary Health Care, Specialized Health Care, and Hospital Health Care in Brazilian regions. Brazil, 2007 to 2020

sPRIMARY HEALTH CARE

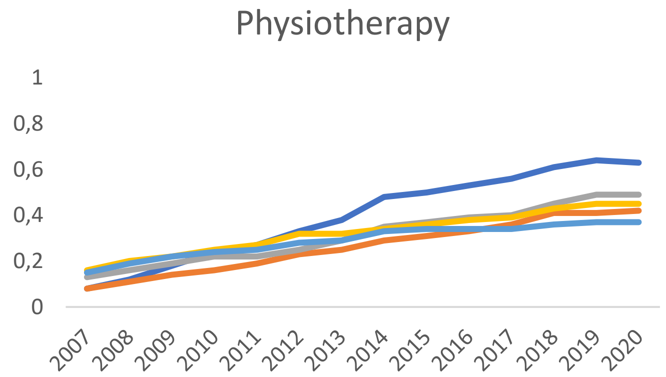

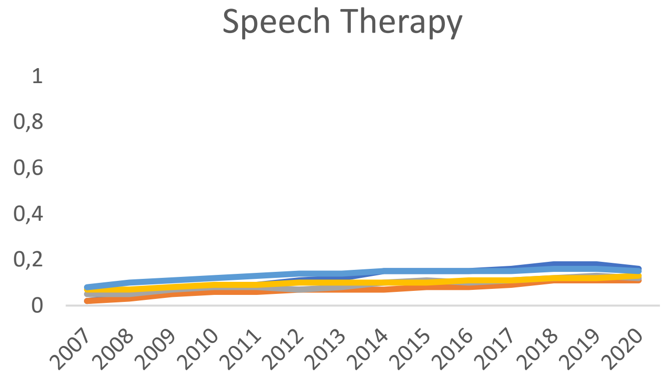

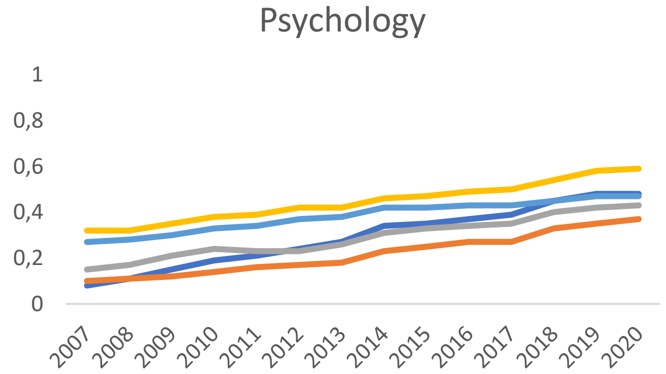

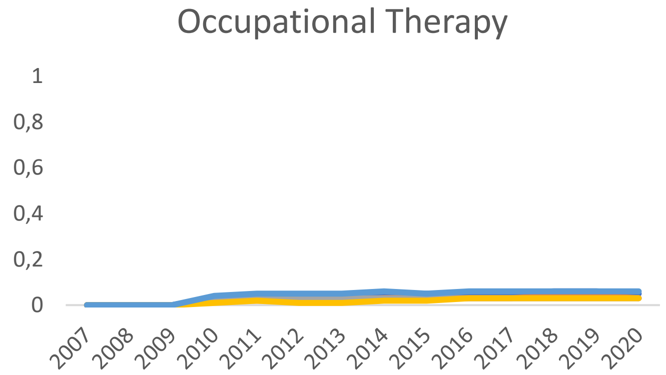

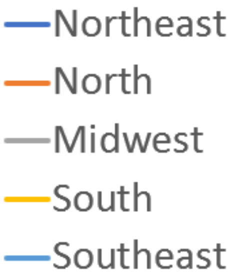

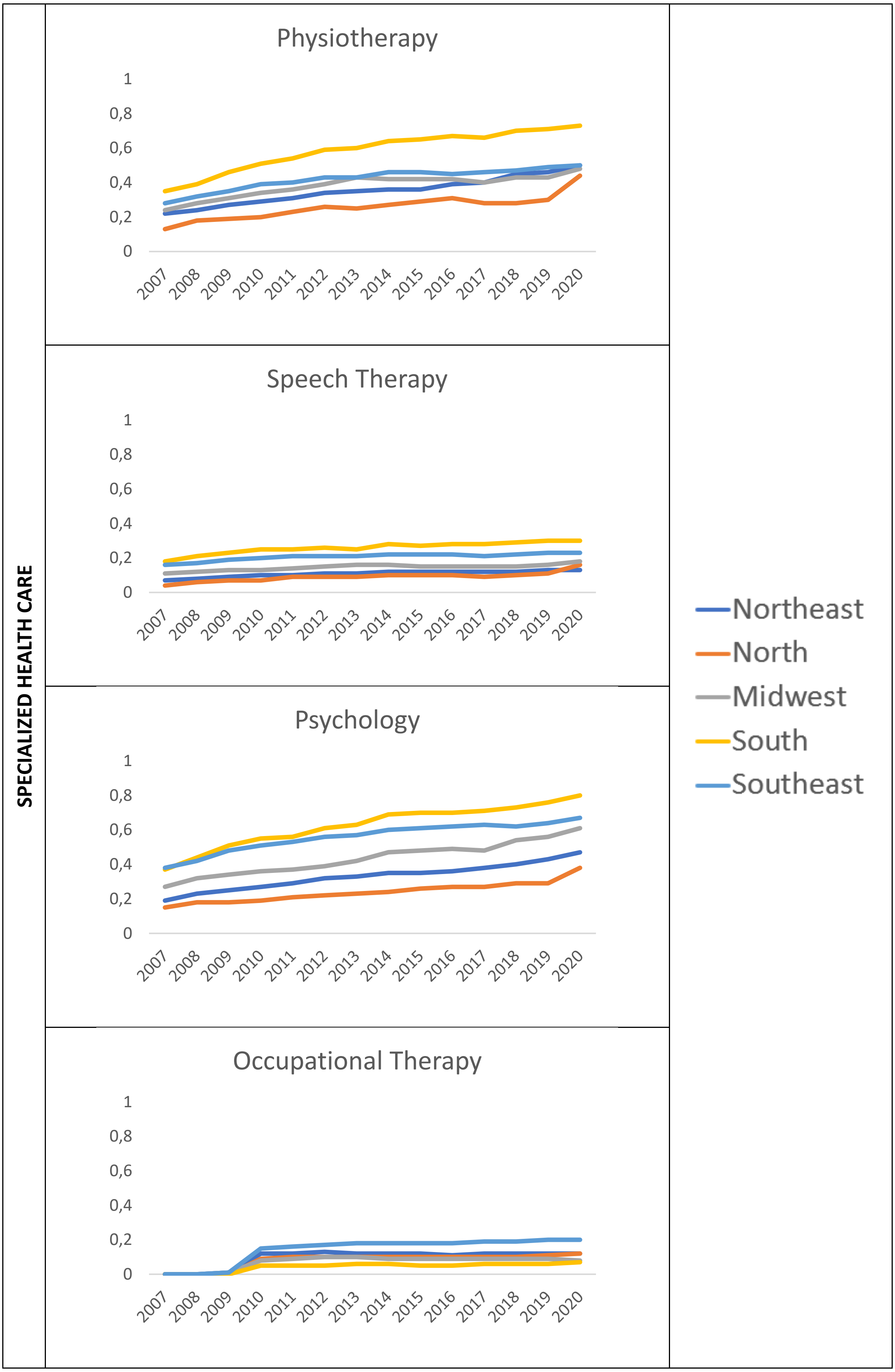

SPECIALIZED HEALTH CARE
Physiotherapy
Speech Therapy
Psychology
Occupational Therapy
1
0,8
0,6
0,4
0,2
0
2007
2008
2009
2010
2011
2012
2013
2014
2015
2016
2017
2018
2019
2020
Northeast
North
Midwest
South
Southeast

HOSPITAL HEALTH CARE

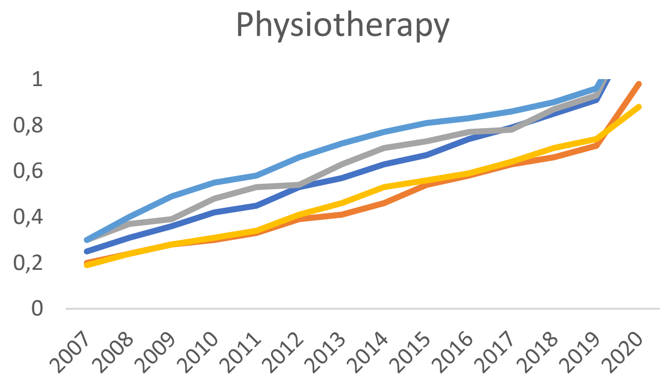

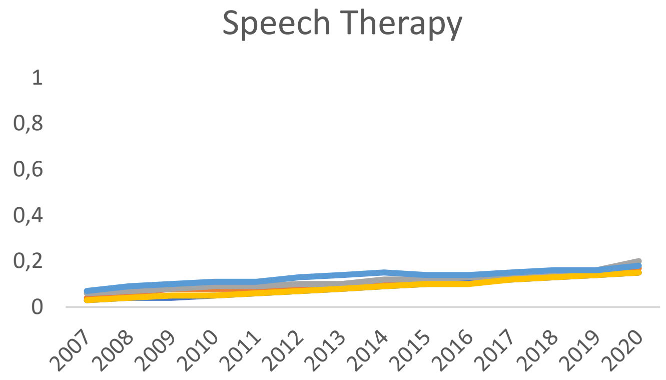

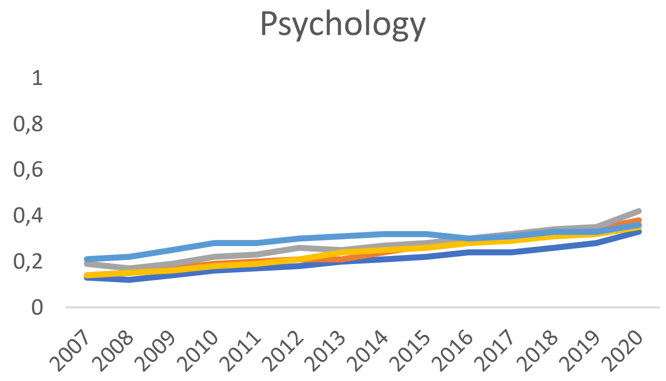

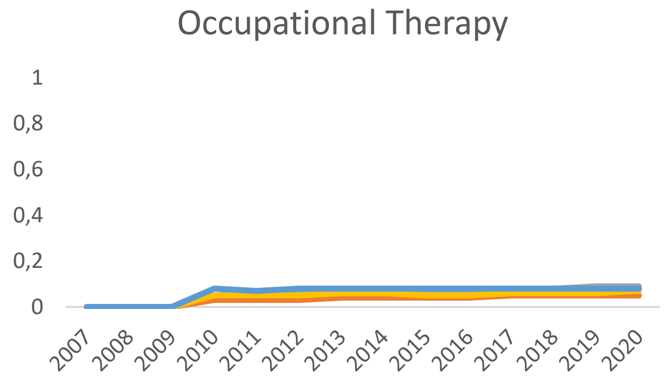

Northeast
North
Midwest
South
Southeast
